# Effects of a single dose of a betalain-rich concentrate on determinants of running performance and recovery muscle blood flow: a randomized, triple-blind, placebo-controlled, crossover trial

**DOI:** 10.1007/s00421-025-05738-w

**Published:** 2025-02-24

**Authors:** Steven Vitti, Michael Bruneau, Leah Bisgrove, Samuel Grey, Sarah Levine, Craig Mattern, Justin Faller

**Affiliations:** 1 Health Sciences Department, Drexel University, Philadelphia, PA USA; 2 Department of Kinesiology, Sport Studies & Physical Education, SUNY Brockport, Brockport, NY USA

**Keywords:** Running economy, Beetroot, Betalains, Blood flow

## Abstract

**Purpose:**

This study aimed to examine the effects of a single dose of a betalain-rich concentrate (BRC) on determinants of running performance and recovery lactate clearance.

**Methods:**

In this a randomized, triple-blind, placebo-controlled, crossover trial, 17 male recreational runners (Age: 19.0 ± 1.0 years; Height: 176.8 ± 6.2 cm; Weight: 70.62 ± 8.1 kg; Body fat: 12.8 ± 0.03%; VO_2max_: 58.9 ± 8.6 ml/kg/min) consumed an acute dose of a BRC (100 mg) or a placebo (100 mg of dextrose; CON) and performed a running economy protocol (RE) at velocities corresponding to 60% and 80% of maximal oxygen consumption (VO_2max_) followed by a VO_2max_ test. Both exercise and recovery skeletal muscle oxygenation (SmO_2_) were assessed via near-infrared spectroscopy (NIRS), and recovery lactate was obtained.

**Results:**

BRC resulted in lower heart rate (HR) at velocities corresponding to 80% of VO_2max_ (*p* = 0.05) and maximum HR (HR_max_; *p* = 0.01), and a lower rating of perceived exertion (RPE) at velocities corresponding to 60% of VO_2max_ (*p* = 0.02) when compared to CON. BRC also improved post-exercise SmO_2_ at 3 (*p* = 0.05), 4 (*p* = 0.02), and 5 min (*p* = 0.01) but not lactate clearance (*p* > 0.05). BRC did not significantly improve RE or VO_2max_ (*p* > 0.05).

**Conclusions:**

A single dose of BRC did not enhance RE or VO_2max_. However, the observed improvements in exercise HR, RPE, and SmO₂ suggest that BRC may confer cardiovascular benefits for exercise and recovery.

## Introduction

Exercise-induced increases in metabolic demands lead to elevated production of reactive oxygen species (ROS). Oxidative stress is defined as an imbalance of the production of ROS and the body’s antioxidant defenses, resulting in a state that favors the production of oxidants (Powers et al. 2020). The production of ROS is dependent upon exercise duration and intensity. While an acute bout of exercise at a moderate intensity can disturb redox balance, high-intensity exercise leads to an excessive production of ROS, which can induce muscular fatigue (Powers and Jackson 2008; Wang et al. 2021). Specifically, ROS have been shown to alter calcium handling at the sarcoplasmic reticulum (Wang et al. 2021), alter muscle fiber excitability by decreasing Na + /K + pump activity (Powers et al. 2020), and reduce myofibril sensitivity to calcium (Cheng et al. 2016), reducing force production. In addition, ROS reduces nitric oxide (NO) availability, causing endothelial dysfunction, which can decrease blood flow to working tissue (Esatbeyoglu et al. 2015).

Beetroot has garnered significant attention for its ergogenic properties. The mechanisms underpinning beetroot’s performance improvements are largely mediated through its rich nitrate content (Jones et al. 2021). When ingested, nitrate is converted to NO downstream, which has been shown to increase performance (Wilkerson et al. 2012), exercise efficiency (Lansley et al. 2011), and fatigue resistance (Cuenca et al. 2018) acutely. However, red beetroot contains other various bioactive components, including betalains (Clifford et al. 2015). Betalains are bioactive pigments found in vegetables such as red beetroot that have known antioxidant and anti-inflammatory properties (Nirmal et al. 2023). The health benefits of betalains have been explored for years. Particularly, betalains alone have been shown to reduce inflammation (Pietrzkowski et al.), improve vascular function (Nemzer et al. 2020), and alleviate oxidative stress (Nilesh Prakash et al. 2023). However, despite sharing many characteristics of popular plant compounds researched in sport and exercise, such as anthocyanins (Kimble et al. 2023), little attention has been given to the potential ergogenic properties of betalains independent of the nitrates included in most beetroot supplements.

To date, only three studies have examined the effects of a betalain-rich concentrate (BRC) free of nitrates on exercise performance. Using sub-chronic doses of a BRC (i.e., 7-day loading schemes), researchers reported improvements in running performance (Van Hoorebeke et al. 2016; Montenegro et al. 2017), cycling performance and economy (Mumford et al. 2018), and enhanced recovery performance (Montenegro et al. 2017). Of the studies conducted, two have reported reductions in post-exercise lactate dehydrogenase (Van Hoorebeke et al. 2016) and creatine kinase (Montenegro et al. 2017), common markers of muscle damage, following running time trials, and modest improvements in blood flow as measured by flow-mediated dilation (FMD; Mumford et al. 2018) following BRC supplementation. The purported mechanisms for these findings were collectively linked to BRC’s biological properties and ability to preserve muscle fiber integrity and support NO availability.

To date, however, no study has examined BRC’s potential on determinants of running performance such as maximal oxygen consumption (VO_2max_) and running economy (RE). Moreover, whether a single dose rather than a short loading period could effectively improve running and recovery blood flow is unknown. Therefore, the purpose of this study was to examine the effects of a single dose of a BRC on factors associated with running performance such as VO_2 max_ and RE. Recovery muscle oxygenation and lactate were assessed to determine if BRC-induced improvements in blood flow would alter post-exercise lactate kinetics. Based on the previous work demonstrating the efficacy of other bioactive pigments on exercise and recovery and the limited work on BRC, we hypothesized that a single dose of 100 mg of a BRC would improve determinants of running performance and recovery blood flow and lactate clearance.

## Methods

### Participants

Seventeen male recreational runners (Age: 19.0 ± 1.0 yrs; Height: 176.8 ± 6.2 cm; Weight: 70.6 ± 8.1 kg; Body fat: 12.8 ± 0.03%; VO_2max_: 58.9 ± 8.6 ml/kg/min) were recruited to participate in this study. To be eligible for this study, participants must have run at least 3 days per week and recorded a minimum of 20 miles per week. A pre-participatory screening (pg. 1 of PAR-Q +) assessed physical activity readiness and medication use, and a brief activity questionnaire was used to determine eligibility. Individuals were excluded if they: (1) had any musculoskeletal injuries that could be made worse by participating in this study (e.g., sprain or strain of lower limb); (2) had documented medical conditions (i.e., hypertension, asthma, heart disease, diabetes) or exhibited signs and symptoms of medical conditions; (3) were taking any medication that would interfere with the interpretation of our results. These included but were not limited to anti-inflammatory drugs, antibiotics, anti-hypertensives, and any medicine controlling the digestive system. All procedures were approved by the Institutional Review Board at SUNY Brockport in accordance with the Declaration of Helinski.

### Study design

This study employed a randomized, triple-blind, placebo-controlled, crossover, counterbalanced design. A triple-blind design ensured that participants, researchers, and statistician remained unaware of treatment allocation until after data analysis was completed. All eligible participants reported to the laboratory at approximately the same time and day for each visit. During the familiarization visit (visit 1), participants provided written consent and received an overview of the study protocol. Afterward, anthropometric assessments were obtained, and a VO_2max_ test was conducted. The VO_2max_ test was used to assess each participant’s aerobic capacity and determine running economy velocities to be used during subsequent testing sessions. Before leaving the lab, participants were instructed to arrive at the lab for visits 2 and 3 following an overnight fast of at least 10 h, to avoid strenuous activity and alcohol consumption 24 h before testing, and avoid caffeine consumption 12 h before testing. Additionally, participants were asked to abstain from taking over-the-counter anti-inflammatory drugs (i.e., Advil or Tylenol) 24 h prior to testing. The first testing session (visit 2) was scheduled for no less than 48 h after visit 1.

### Procedures

**Fig. 1:**
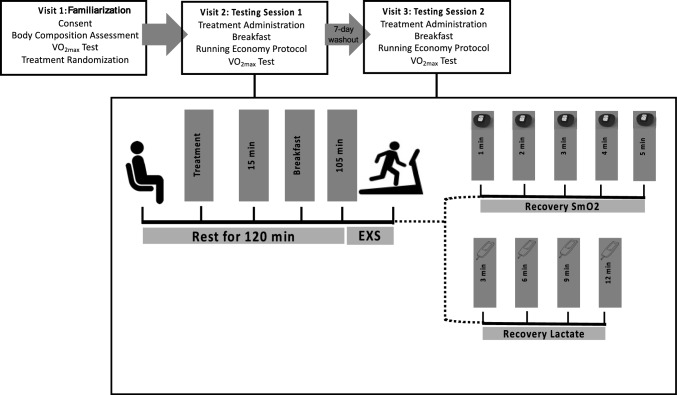
Visual representation of the study timeline. *SmO*_*2*_ skeletal muscle oxygenation, *VO*_*2max*_ maximal oxygen uptake, *EXS* exercise testing

For a visual representation of the study timeline, see Fig. 1. Upon arrival to the laboratory, participants were given their predesignated supplement, which consisted of either 100 mg of a BRC or a placebo and were asked to rest quietly in a seated position for 15 min. Afterward, each participant was given a standardized breakfast containing a Clif Bar (250 kcal; 5 g FAT; 45 g CHO; 9 g PRO), medium-sized banana (105 kcal; 0 g FAT; 27 g CHO; 1 g PRO), and 500 ml of water. Once consumed, each participant was asked to sit for an additional 105 min. During this time, the participants were fitted with a heart rate (HR) monitor (Polar Unite Heart Rate Sensor, Polar, Finland) and a near-infrared spectroscopy device (NIRS; Moxy Monitor; Minnesota, MN) placed on the belly of the vastus lateralis of their dominant leg, approximately 12 cm above the knee, which was used to record skeletal muscle oxygenation (SmO_2_). After the rest period, blood lactate (LAC) was obtained via a capillary finger stick (Lactate Plus Meter, Nova Biomedical, Waltham, MA). Once the participants were ready to begin the RE protocol, they were asked to proceed with a warmup protocol, which consisted of 5 min of treadmill running at a self-selected pace. Upon completion of the warmup, the participants were fitted with a mouthpiece that allowed the collection and analysis of respiratory gasses during exercise.

Once ready, participants began the RE protocol and, when completed, were given a 5 min break where they walked on the treadmill at 5 kph, then commenced with the VO_2max_ test, which mirrored that of Visit 1, only the test began at the velocity corresponding to 80% of their VO_2max_. After completing the VO_2max_ test, participants were instructed to rest in a seated position, and SmO₂ was recorded every minute for a total of 5 min. Recovery LAC levels were measured 3, 6, 9, and 12 min after the test. The subsequent testing session followed the same procedures as the first, but participants received the alternate treatment to what they received during visit 2. The final testing session (visit 3) was scheduled after a 7-day washout to allow full recovery from exercise and prevent any carryover effects from the previous treatments.

### Running economy protocol

The RE consisted of running at velocities corresponding to 60% (vVO2_60%_) and 80% (vVO2_80%_) of their VO_2 max_ for 5 min at each velocity, with no rest given between each velocity. Respiratory exchange ratio (RER), rating of perceived exertion (RPE), the volume of oxygen consumed (VO_2_), and HR were recorded during the last 30 s of each velocity. SmO_2_ was recorded every 2 s during the last 30 s at each velocity using training software (PerPRO Studio©, Dynastream Innovations Inc., Canada), and the average of the values were recorded as SmO_2_ for each velocity.

### VO_2max_ measurement

VO_2max_ testing was performed on a motorized treadmill (Trackmaster TMX58, Trackmaster Treadmills, Newton, KS) at an incline of 1%. Following a warmup consisting of a 3 min walk at 6 kph, the protocol was initiated at a speed of 8 kph and was increased 1 kph every 1 min until a speed of 16 kph was reached. After this point, the incline increased by 2% every 1 min until volitional exhaustion occurred. HR, RER, RPE, and VO_2_ were recorded as the highest values observed in the last 30 s of each stage. Breath-by-breath analysis was recorded every 15 s via indirect calorimetry (TrueOne 240, Parvo Medics, Murray, UT), and all VO_2_ values were expressed in milliliters per kilogram per minute (ml/kg/min). SmO_2_ was recorded as an average of 30 s at the end of each stage. The test was terminated upon volitional exhaustion and test duration, RPE, SmO2, and VO_2 max_ were recorded.

### Treatment randomization

The treatments consisted of a single dose of a BRC supplement consisting of 100 mg (2 X 50 mg) of a freeze-dried beetroot concentrate containing a standardized 25% betalains, or a placebo consisting of 100 mg (2 X 50 mg) of dextrose (CON). A 100 mg dose was selected based on findings that indicated that doses above 50 mg resulted in greater reductions in pro-inflammatory markers (Pietrzkowski et al.). An individual not participating in data collection or analysis was responsible for randomization and blinding the participants and researchers to the treatments. All treatments were supplied in identical capsules to ensure blinding status. All treatments were randomized and allocated triple-blinded, and counterbalancing was used to mitigate potential order effects.

### Statistical analysis

A preliminary power analysis was conducted for each dependent variable with G*Power version 3.1 to detect a medium effect size with 80% power. Seventeen participants were recruited and enrolled to meet this criterion successfully. Unless otherwise noted, descriptive statistics (means ± standard deviations) were computed for all study variables. A 2 (condition) X 4 (time) repeated measures (condition X time) analysis of variance (ANOVA) was used to determine mean differences in LAC. A 2 (condition) X 5 (time) repeated measures (condition X time) ANOVA was used to determine mean differences in recovery SmO_2_. Separate 2 (condition) X 3 (time) repeated measures (condition X time) ANOVA were used to determine mean differences in exercise RPE, HR, RER, and SmO_2_.

Data screening and cleaning procedures were performed to assess the presence of statistical outliers based on a standardized z-score of >  ± 3.33. Exercise SmO_2_ values were not transmitted by the NIRS device for one participant during one of the exercise sessions, resulting in a data loss. Multiple imputation procedures were employed for missing data before assessing our central hypotheses with inferential statistical procedures. The basic assumption of normality was assessed and verified for all dependent variables with skewness and kurtosis statistics, confirmed with measures <  ± 3.33 standard deviations below the mean distribution. Mauchly’s Test of Sphericity validated the covariance matrix for a time in our repeated measures factor. When the sphericity assumption was violated, Greenhouse–Geisser adjustments were employed. Significant main and interaction effects were assessed *post-hoc* with pairwise comparisons and simple effects tests with Bonferroni adjustments to control for the familywise error rate. All statistical analyses were performed with the SPSS version 27.0, and alpha levels were set a priori to *p* < 0.05.

## Results

### Oxygen uptake

There were no statistically significant differences in VO_2max_ (BRC = 60.04 ± 9.06 ml/kg/min; CON = 59.21 ± 11.11 ml/kg/min; *p* > 0.05) or VO_2_ at vVO2_60%_ (BRC = 38.51 ± 4.78 ml/kg/min; CON = 37.37 ± 4.27 ml/kg/min; *p* > 0.05) or vVO2_80%_ (BRC = 49.14 ± 6.87 ml/kg/min; CON = 48.57 ± 7.52 ml/kg/min; *p* > 0.05) between BRC and CON.

### Rating of perceived exertion

**Fig. 2:**
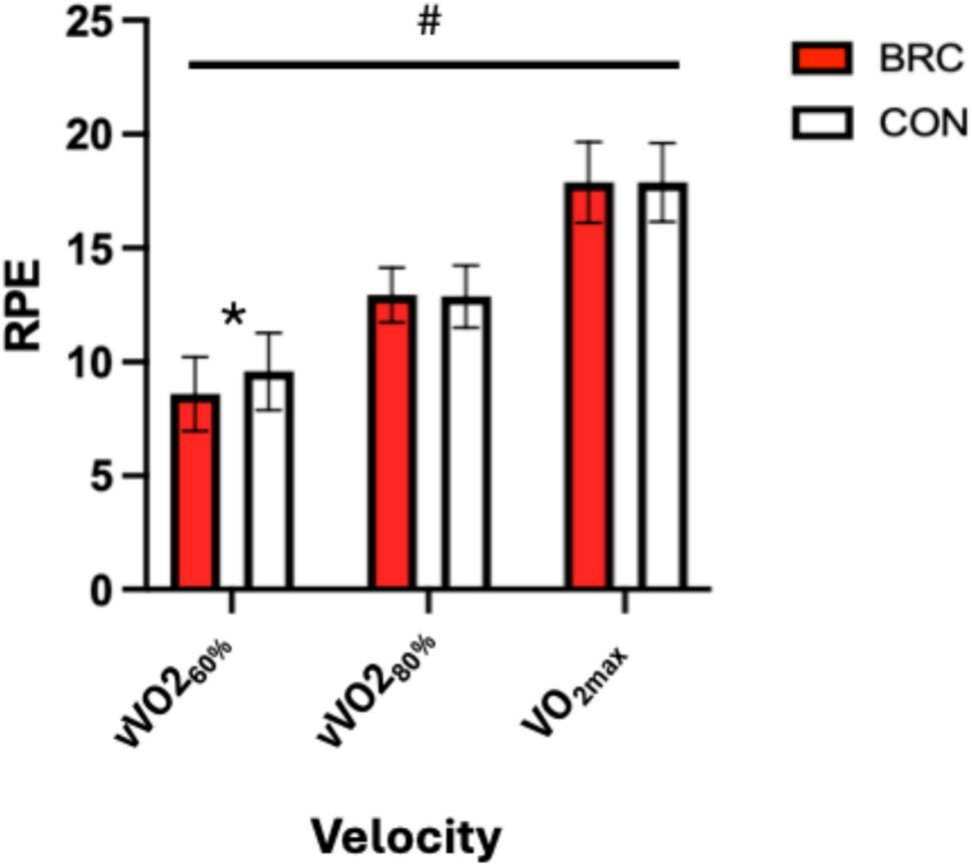
Mean ± standard deviation of RPE at each running economy velocity and VO_2max_. *Denotes a significant between conditions (BRC vs. CON). # Denotes a significant difference over time independent of condition. *BRC* betalain-rich concentrate, *CON* control, *vVO2*_*60%*_ velocity at 60% VO_2max_, *vVO2*_*80%*_ velocity at 80% VO_2max_, *RPE* rating of perceived exertion

RPE significantly increased with exercise intensity (*p* = 0.00). Additionally, there was a significant [supplement X time] interaction (*p* = 0.05; η^2^ = 0.17). A post hoc analysis revealed the BRC treatment reported significantly lower RPE (*p* = 0.02) than the CON (see Fig. 2) at vVO2_60%_. There were no significant differences in RPE at vVO2_80%_ or VO_2max_ between treatments (*p* > 0.05).

### Respiratory exchange ratio

BRC did not confer any significant changes in substrate utilization for either the running economy velocities or VO_2 max_ when compared to CON (*p* > 0.05).

### Exercise skeletal muscle oxygenation

A significant time effect was observed for SmO_2_ (*p* = 0.00, η^2^ = 0.87). Specifically, SmO_2_ at vVO2_60%_ (49.29 ± 11.13%; CON = 47.18 ± 12.63%) were significantly greater than vVO2_80%_ (36.76 ± 12.41%; CON = 34.65 ± 13.17%; *p* = 0.00) and VO_2max_ (24.72 ± 12.85%; CON = 18.47 ± 7.36% *p* = 0.00); and SmO_2_ was significantly lower at vVO2_80%_ when compared to values at VO_2max_ (*p* = 0.00). This is an expected physiological response to an increased intensity. No other significant differences were observed for SmO_2_ during exercise.

### Heart rate

**Fig. 3:**
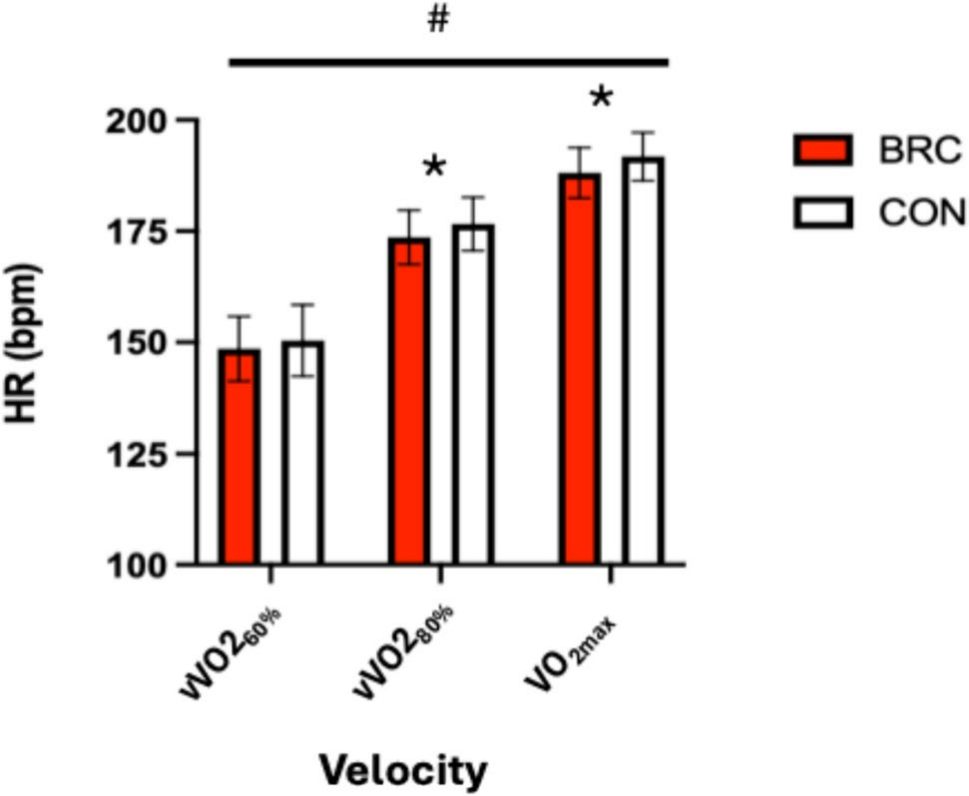
Mean ± standard deviation of HR at each RE velocity and VO2max. * Denotes a significant between conditions (*p* < 0.05; BRC vs. CON). #Denotes a significant difference over time independent of condition (*p* < 0.05). *BRC* betalain-rich concentrate, *CON* control; *vVO2*_*60%*_ velocity at 60% VO_2max_, *vVO2*_*80%*_ velocity at 80% VO_2max_, *HR* heart rate

A significant treatment (*p* = 0.04, η^2^ = 0.23) was observed for HR. A pairwise comparison revealed that HR was lower at vVO2_80%_ (*p* = 0.05) and VO_2 max_ (*p* = 0.01) in the BRC treatment compared to CON. The analysis reported a significant time effect (*p* = 0.00; η^2^ = 0.94) for HR. The notable time effect is due to the significant rise in heart rate with increasing intensity, which aligns with the expected physiological response (see Fig. 3).

### Recovery skeletal muscle oxygenation

**Fig. 4:**
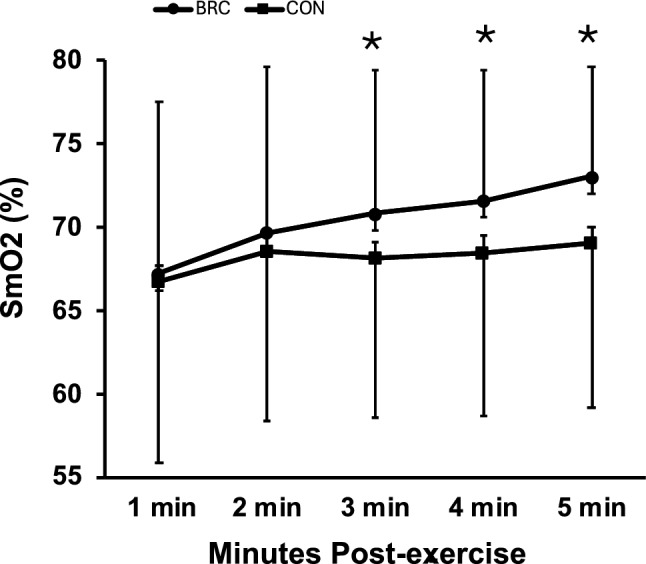
Mean ± standard deviation of SmO2 over time. *Denotes a significant between conditions (*p* < 0.05; BRC vs. CON). *BRC* betalain-rich concentrate, *CON*  control, *SmO*_*2*_ skeletal muscle oxygenation

A significant [treatment X condition] interaction was observed for recovery SmO_2_ (*p* = 0.05, η^2^ = 0.17). Post hoc analysis revealed that the BRC treatment resulted in greater SmO_2_ at min 3 (*p* = 05), min 4 (*p* = 0.02), and min 5 (*p* = 0.01) post-exercise when compared to the CON treatment (see Fig. 4).

### Recovery lactate

**Fig. 5:**
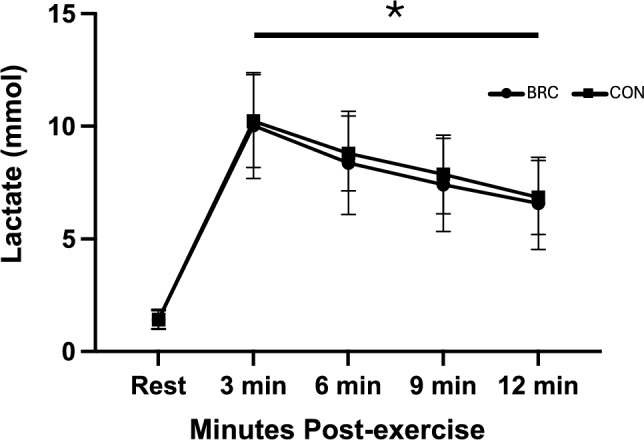
Mean ± standard deviation of LAC over time. *Denotes a significant time effect independent of condition (*p* < 0.05). *BRC*  betalain-rich concentrate, *CON* control

Data for recovery LAC is reported in Fig. 5. A significant time effect was reported for recovery LAC (*p* = 0.00; η^2^ = 1.00). LAC was significantly elevated post-exercise compared to baseline and remained elevated for the duration of the recovery period. No other main effects or interactions were observed.

## Discussion

The purpose of this study was to examine the effects of a single dose of a BRC on determinants of running performance, exercising and recovery SmO_2_, and LAC clearance. This work adds to the limited evidence for the potential ergogenic properties of a BRC. The findings of the current study were that a single dose of 100 mg of a BRC: 1) did not improve running economy, VO_2max_, or maximal O2 extraction during maximal effort, 2) reduced RE RPE at vVO2_60%_, 3) reduced HR_max_ and HR at vVO2_80%_; 4) improved post-exercise muscle oxygenation, and 4) did not influence post-exercise LAC clearance when compared to a CON.

Contrary to our hypothesis, we found no improvements in VO_2max_ or RE following a single dose of BRC. To our knowledge, this is the first study to examine a single dose of BRC and performance-related variables and not performance per se. To date, only a small handful of studies have examined the ergogenic effects of a BRC. In a study of recreational runners by Van Hoorebeke et al. (2016), researchers reported that six days of 100 mg BRC and 50 mg immediately prior to exercise testing improved 5 km time trial (TT) performance, but not 30-min of submaximal running. Using an identical dosing strategy, Montenegro et al. (2017) reported that BRC reduced time to completion by 3% in both 10 km TT and recovery 5 km TT performance but did not improve 40-min of submaximal cycling in a group of competitive triathletes. Most recently, Mumford et al. (2018) observed that one week of a BRC increased absolute power output during a 30-min TT cycling performance in well-trained cyclists. Several mechanisms have been proposed to explain the observed improvements in performance, all of which are attributed to the antioxidant and anti-inflammatory properties of betalains. Specifically, BRC may preserve the integrity of muscle fibers during exercise by attenuating inflammation and/or by preserving NO synthesis via the scavenging of reactive oxygen and nitrogen species, which could increase blood flow to working skeletal muscle (Richards et al. 2018) and potentially reduce the energetic cost of muscle contractions (Bailey et al. 2010). The former mechanism is supported by the reductions in markers of muscle damage following a 5 km TT and 10 km TT run in the Van Hoorebeke and Montenegro studies, respectively. Interestingly, though, Mumford et al. (2018) found that one week of a BRC failed to attenuate the inflammatory response following a 30-min cycling TT. One possible explanation for the disparity in findings could be the intensity of the exercise task employed. Specifically, it is known that inflammatory cytokines and ROS are produced in larger quantities at higher intensities. Thus, the exercise task employed by Mumford et al. (2018) may not have been adequate to alter inflammation in a way that would inhibit performance. In the current study, however, a single dose of BRC failed to alter running economy at speeds considered to be high intensity or improve VO_2 max_ during a graded exercise task.

Regarding the latter mechanism, the current study found SmO_2_ was significantly greater at 3 min, 4 min, and 5 min post-exercise following BRC. Moreover, we observed significantly lower RPE at vVO2_60%_ and lower HR at vVO2_80%_ as well as HR_max_ following BRC, which is somewhat in line with the reductions in HR and RPE observed by Van Hoorebeke et al. (2016) during 30 min of submaximal running following BRC. Nemzer et al. (2020) reported significant increases in FMD, a measure of endothelial function, with corresponding increases in bioavailable NO, 2 h following a single 50 mg dose of a BRC. Similarly, a single dose of a betalain-rich dragon fruit powder containing ~ 33 mg of betalains improved several markers of vascular function in young, healthy adults (Cheok et al. 2022). Lastly, Mumford et al. (2018) reported a slight increase in FMD in the BRC condition. Considering the significantly lower HR at higher intensities, our findings could reflect NO-induced cardiovascular adjustments via the antioxidant properties of BRC. However, it is important to stress caution when interpreting and comparing the findings of the current study due to the variability in supplementation and dosing strategies employed in previous work.

Regarding the overall absence of findings in VO_2max_ or RE, it is likely that longer administration doses are warranted. All three BRC previous studies reported herein employed a sub-chronic dosing scheme that consisted of 100 mg/day for six days, followed by a pre-exercise dose of 50 mg. Betalains have been shown to activate the Nrf2 pathway, an important regulator of the endogenous antioxidant system (Krajka-Kuźniak et al. 2013). Therefore, it is plausible to suggest that BRC antioxidant and anti-inflammatory effects may be mediated through Nrf2, not BRC innate antioxidants per se. A recent investigation by Wangdi and colleagues (2021) found that supplementation with tart cherry juice rich in polyphenols for 7 days prior and 2 days following 300 eccentric contractions of the knee flexors attenuated decrements in strength loss. Notably, the authors reported corresponding increases in glutathione peroxidase 1, a cellular antioxidant enzyme, likely expressed via the Nrf2-antioxidant response element signaling pathway (Wangdi et al. 2022). Future studies are warranted to explore whether the benefits of BRC are conferred by bolstering endogenous antioxidant capacities.

Blood flow to and from working tissue, diffusion gradient, and mitochondrial function collectively influence LAC production, removal, and metabolism (Emhoff and Messonnier 2023). Therefore, it is reasonable to suggest that improvements in blood flow would influence LAC kinetics during and following exercise (Cook et al. 2015). However, despite observing significant increases in SmO_2_ at 3-, 4- and 5-min post-exercise, BRC did not affect post-exercise lactate kinetics. This suggests that the increase in blood flow, as indicated by the change in SmO_2_, was likely insufficient to influence LAC clearance. In a study examining the effects of a blueberry powder, which is rich in anthocyanin, a bioactive compound with known antioxidant properties, following 2 days and 4 days of administration, researchers reported improved lactate kinetics following four days of blueberry supplementation only (Brandenburg and Giles 2019). This may indicate that longer supplementation periods are needed to experience performance benefits. Methodological differences may also explain the difference in our findings. The authors measured lactate following an 8-km TT, while the current study assessed LAC clearance following maximal testing. Consequently, a possible explanation for the discrepancy in our findings could be that the acidosis that coincides with lactate levels achieved herein was enough to disrupt acid–base balance and thus inhibited mitochondrial respiration, an important variable for LAC clearance (Chatel et al. 2016).

A major strength of the current study is its novelty. This is the first to examine the potential effects of a single dose of a BRC on exercise. We believe the current work significantly contributes to the limited body of knowledge pertaining to BRC’s ergogenic capacities and begins to shed light on optimal dosing strategies required to improve performance. Regarding limitations, due to financial constraints, plasma betalains and NO were not measured in this study. Moreover, markers of inflammation and oxidative stress were not quantified herein. Thus, we cannot say our protocol sufficiently perturbed redox homeostasis in a way that would reflect determinants in performance, nor can we definitively conclude any improvements were attributed to BRC antioxidants and antioxidant properties. In addition, this was the first study to examine the effects of a single dose of a BRC, which may not be sufficient to detect any measurable benefit in performance. Therefore, prolonged dosing strategies may be required to see any benefits in performance. However, since long-term suppression of exercise-induced free radical production could hinder adaptations (Powers et al. 2020), such approaches may ultimately be counterproductive and should be approached with caution. Additionally, diet logs were not collected as part of this study. Considering that dietary habits, particularly fruit and vegetable consumption, are linked to antioxidant status, we believe this to be a limitation. In addition to the aforementioned limitations, future studies should consider examining both single and sub-chronic dosing strategies on high-intensity exercise performance. Considering the relationship between exercise intensity and duration and disturbances in redox homeostasis, BRC may hold promise for exercise requiring higher intensities and longer durations. Finally, this study only consisted of male participants, limiting the generalizability of the findings. Thus, all future studies should include female participants, especially given their underrepresentation in the literature.

In conclusion, a single dose of a BRC significantly reduced HR and RPE at submaximal speeds and HR_max_ but failed to improve RE and VO_2max_ in a group of recreational runners. Improvements in SmO_2_ were observed in the latter minutes of exercise recovery, suggesting BRC may confer some cardiovascular benefits; however, these observations were observed in the absence of any difference in recovery LAC.

## Data Availability

The primary data for this study is available from the authors upon direct request.

## Abbreviations

ANOVA: Analysis of variance
BRC: Betalain-rich Concentrate
CON: Control
FMD: Flow-mediated dilation
LAC: Lactate
NO: Nitric oxide
RE: Running economy
RER: Respiratory exchange ratio
ROS: Reactive oxygen species
RPE: Rating of perceived exertion
SmO_2_: Skeletal muscle oxygenation
TT: Time trial
VO_2_: Oxygen uptake
VO_2max_: Maximal oxygen uptake
vVO2_60%_: Velocity at 60% VO_2max_
vVO2_80%_: Velocity at 80% VO_2max_
